# Treatment needs and expectations for Fabry disease in France: development of a new Patient Needs Questionnaire

**DOI:** 10.1186/s13023-019-1254-7

**Published:** 2019-12-04

**Authors:** Esther Noël, Bertrand Dussol, Didier Lacombe, Najya Bedreddine, Alain Fouilhoux, Pierre Ronco, Delphine Genevaz, Soumeya Bekri, Albert Hagège, Frédérique Dupuis-Siméon, Valérie Derrien Ansquer, Dominique P. Germain, Olivier Lidove

**Affiliations:** 1 Strasbourg University Hospital, Strasbourg, France; 2 Conception Hospital, Marseille, France; 3 Bordeaux University Hospital, INSERM U1211, Bordeaux University, Bordeaux, France; 4 Association des Patients de la Maladie de Fabry (APMF), Marsannay la Côte, France; 5 Lyon University Hospital - Lyon Civil Hospital, Lyon, France; 6 Tenon Hospital, Paris, France; 7 Vaincre les Maladies Lysosomales (VML), Massy, France; 8 Rouen University Hospital, Rouen, France; 9 Georges Pompidou European Hospital, Paris, France; 10 Amicus Therapeutics, La Défense, France; 11 A+A Research, Lyon, France; 12 French Referral Center for Fabry disease, Division of Medical Genetics and INSERM U1179, University of Versailles, Paris-Saclay University, Montigny, France; 13 La Croix St Simon Hospital, Paris, France

**Keywords:** Fabry disease, Patient association, Patient-reported outcome measure, Patient self-reported tool, Patient Needs Questionnaire, Psychometric analysis, Lysosomal disorders

## Abstract

**Background:**

Fabry disease (FD) is a rare, X-linked, inherited lysosomal disease caused by absent or reduced α-galactosidase A activity. Due to the heterogeneity of disease presentation and progression, generic patient-reported outcome (PRO) tools do not provide accurate insight into patients’ daily lives and impact of disease specific treatments. Also, the French National Health Authority, (HAS) actively encourages a patient-centric approach to improve the quality of care throughout the patient journey. In response to this initiative, we aimed to develop and validate a specific, self-reported, Patient Needs Questionnaire for people living with Fabry disease to appraise patient needs and expectations towards their treatment (PNQ Fabry). This endeavour was led with the help of French patient associations (APMF & VML) and dedicated expert centres. PNQ Fabry was developed according to the FDA/EMA methodologies and best practices for the development of PRO tools in rare diseases. Our approach comprised of three steps, as follows: concept elicitation and item generation, item reduction, and final validation of the questionnaire through a two-stage survey.

**Results:**

Intrinsic and extrinsic reliability was established, using a validated benchmark questionnaire. With the invaluable help of patient associations, we recruited a satisfactory population in this rare disease setting, to ensure robust participation to validate our PNQ (final number of questionnaires: 76). At the end of the process, a 26-item patient-reported questionnaire was obtained with excellent psychometric properties, exhibiting very satisfactory measurement outcomes for reliability and validity. The results of this initiative demonstrate that the PNQ Fabry is accurate, suitable and tailored to FD patients, as it addresses themes identified during patient interviews, that were further validated through statistical analyses of quantitative surveys. An ongoing phase IV study is using this tool.

**Conclusion:**

We believe the PNQ Fabry will be a reliable and insightful tool in clinical practice, to improve patient management in FD.

## Introduction

Caring for people with Fabry Disease (FD) requires careful, lifelong monitoring to manage the multisymptomatic effects of the disease. These patients live with chronic cardiac, renal and neurological problems that reduce life expectancy and require substantial supportive therapy. These problems diminish quality of life and cause significantly higher psychological distress than other chronic conditions [1–4]. Although current enzyme replacement therapies (ERT) and pharmacological chaperone therapy attenuate the disease progression and alleviate some symptoms, many patients still require ongoing lifestyle modifications and symptomatic medication to manage symptoms and pain [5]. Physicians regularly monitor patients to adapt therapy and control symptoms, whilst aligning with individual patient’s lifestyle needs.

In October 2013 the French National Authority for Health (HAS), published the shared Decision Making report [6]. This report highlights the need to develop a patient-centric approach throughout the patient journey. The objective is to improve the safety and quality of care by combining the patient experience with evidence-based care. This process also provides an environment where patients are encouraged to share their preferences and take an active role in their treatment, and disease management decisions. To do this, the HAS recommended using specific tools to guide patients to prioritise the available therapeutic options according to their quality of life preferences.

Measuring individual treatment needs and expectations requires administering a validated instrument when initiating and monitoring therapy. In recent years, specific patient reported outcome (PRO) instruments have been developed to quantify the quality of life and treatment satisfaction for various conditions [7–16]. Yet, to date no such tool is available to assess specific expectations towards the treatment of Fabry disease.

In a clinically heterogeneous disease such as Fabry disease, [2, 3] patient associations can provide meaningful insight into the burden of living with this disease. In France, two associations actively represent Fabry patients, participate in and promote medical research: Association des Patients de la Maladie de Fabry (APMF, apmf-fabry.org) and Vaincre les Maladies Lysosomales (VML, www.vml-asso.org). In a patient-centric approach, involving these associations in developing a patient needs instrument is highly recommended to provide a patient perspective [17, 18].

In this context, the authors identified the need to develop a questionnaire to evaluate and measure treatment expectations for patients living with Fabry disease to heighten the clinical picture and allow physicians to manage the treatment more astutely.

The objective of this research was to develop and validate a specific, self-reported, Patient Needs Questionnaire (PNQ) for people living with Fabry disease to evaluate patient treatment expectations (PNQ Fabry).

## Methods

### Design, quality and ethical conduct

**Fig. 1:**
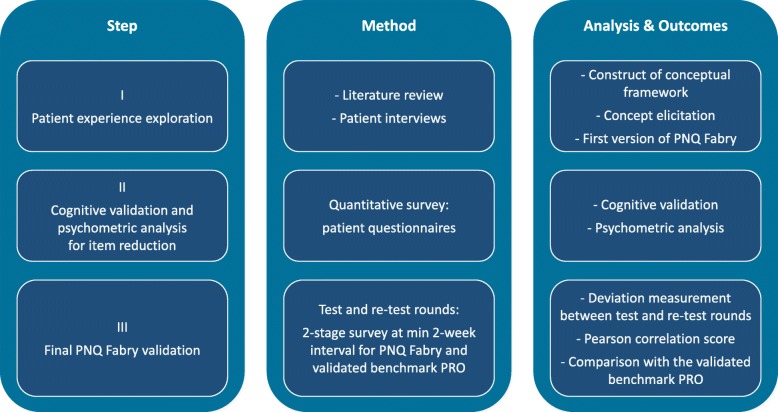
The 3-step PNQ Fabry development and validation process

The PNQ Fabry was developed and validated in a three-step process according to international standards and best practice for developing PRO tools, [19, 20] and Patient Need Questionnaires (PNQ) in the field of rare diseases [21–24]. as illustrated in Fig. 1.

A French board of physicians specialized in treating rare lysosomal diseases worked in close collaboration with the sponsor, Amicus Therapeutics, and two influential patient associations in France, Association des Patients de la Maladie de Fabry (APMF, www.apmf-fabry.org) and Vaincre les Maladies Lysosomales (VML, www.vml-asso.org). All participant experts were highly engaged and actively contributed to the research conception, protocol design, patient recruitment, questionnaire development and transmission over the course of the process.

Each step was conducted in accordance with good research practice and Ethics Committee and Institution Review Board requirements. All research participants received clear and detailed information about their involvement in developing this questionnaire, and their rights as a participant before providing their informed consent and participating in any phase of the process. They were made aware that the results of this research may be published in a peer-reviewed medical journal. Each participant was identified with a unique identifying number and their anonymity, personal or clinical information was protected throughout the process.

### STEP I: concept elicitation

#### Literature review

A detailed review of the literature was performed to clearly understand the disease presentation, symptoms, impact on daily activities, patient perceptions of their disease and treatment. Also, to establish whether existing PRO instruments to measure treatment needs, expectations or satisfaction could be adapted to our research objectives.

The following keywords were used in combination or alone: Fabry disease, orphan diseases, rare diseases, patient reported outcome, PRO instruments, tools, quality of life measurements, treatment satisfaction, benefit and risk measurement, patient need questionnaire, PNQ, patient benefit questionnaire, PBQ, patient benefit index, PBI, assessment of patient needs, patient expectation questionnaire. The systematic search used Google Scholar, Pub-Med, Pub-Med Health, ScienceDirect, ISPOR websites.

Items found in PRO instruments in this search were used in the interview guide to prompt patients during the qualitative in-depth interviews. It also provided additional background information for the interview moderator.

#### Item generation interviews and questionnaire development

Practicing physicians (*n* = 4) from expert centres for metabolic diseases in France and the patient association VML were invited to select eligible patients to participate in the qualitative interviews. Eight participants were planned with a diverse mix of demographics including gender, age, socio-economic status, rural or urban dwellers, employment status, disease severity and current treatment to capture a maximum number of experiences and expectations.

A psycho-sociologist conducted individual telephone interviews guided with open questions about living with Fabry disease and the patient’s treatment experience and expectations. Patients could reply freely, however specific probes determined from the literature review were asked if not spontaneously addressed by the patient. The interviews lasted up to 60 min and were recorded and fully transcribed. Patient verbatims from the interviews were used to generate items (“concept elicitation” step). The data obtained from the literature review and verbatim from the patient interviews were then submitted for semantic analysis with the Atlas-ti software to build a conceptual framework. This means structuring and clustering all information collected into themes / sub-themes / items / verbatim (“dimension” / “root concept” / “concept” / verbatim) (See Additional file 1: Table S1). Any redundancies or nuances were eliminated to reduce the number of items to a manageable number for a survey. These items were then transferred to the first version of the PNQ Fabry for step II quantitative analysis. The items were randomly placed to prevent order or floor effect in patient responses. (Table 1).

**Table 1:** Step II: The first version of PNQ Fabry (58 items): as sent to participants. [*Excerpt from the PNQ Fabry V1*]

	1 – Not at all Important	2 – Slightly Important	3 - Moderately Important	4 - Important	5 – Very Important	6 – Does not apply to me
**How important is it to you that the treatment enables...**						
to be less depressed and restores your zest for life	□	□	□	□	□	□
to feel good every day	□	□	□	□	□	□
to continue to work	□	□	□	□	□	□
not to fear the administration of your treatment	□	□	□	□	□	□
to be able to spend time with your family	□	□	□	□	□	□
to stop the evolution of Fabry disease	□	□	□	□	□	□
not to cause excessive sweating	□	□	□	□	□	□
to reduce pain in the feet	□	□	□	□	□	□
to have less mood swings	□	□	□	□	□	□
not to suffer from side effects or adverse events related to treatment	□	□	□	□	□	□
…	□	□	□	□	□	□
**How important is it to you to have a treatment...**						
that you can pick up at the pharmacy in your town	□	□	□	□	□	□
…	□	□	□	□	□	□

### STEP II: psychometric analysis and cognitive validation

#### Participants and study centres

For this step, participating physicians and key patient organisations, APMF and VML were responsible for recruitment but also reviewing and validating the content of the questionnaire throughout its development. To be eligible, participants were aged at least 16 years, currently receiving enzyme replacement therapy (ERT agalsidase alfa or agalsidase beta) or a chaperone molecule (migalastat), or expected to start treatment within the following 3 months to accurately capture expectations that patients may have before starting treatment.

#### Data collection

Patient associations and a third-party research agency A + A Research sent the questionnaire to eligible and willing participants in either a paper or electronic format. One hundred and fifty questionnaires were provided to ensure at least 65 would be completed and eligible for the statistical calculations. This was expected to be adequate, considering the rarity of this disease and the uncertainty of patient willingness to participate.

Participants were asked to complete three documents. In the first, they rated the level of importance for each item of the PNQ Fabry V1 on a 5-point Likert scale. In the second, they recorded the comprehensibility of each item and the completeness of the item list. In the last, they reported their demographic details and medical history related to their condition.

Data collection was continued until all potential participants had completed or refused the questionnaire.

#### Psychometric and cognitive validation procedures

The third-party research agency performed the analysis and at least two researchers were involved. All statistical analyses were performed with SPSS software.

From the initial list of possible items, a rotational statistical process was employed to refine the number of items to a short-list of most relevant items. Each time an item was deleted the round of statistical analyses was performed until the questionnaire reached an appropriate score to indicate stability and consistency.

#### Content validity

To ensure that the content accurately applied to patient treatment needs and expectations, no semantic dimensions were eliminated during the analysis process. Only items that related to treatment needs and expectations were retained.

To ensure that an item had not been inadvertently omitted, an open question was added for participants to comment. Only those comments related to treatment needs and expectations were included.

The importance of each item was measured by the mean score, the average percentage of participants rating an item as ‘5-Very important’ and the cumulative percentage of participants rating an item as ‘5-Very important’ or ‘4-Important’.

The relevance of each item was evaluated by the percentage of participants choosing “Does not apply to me”.

Analyses were performed on the total sample and by socio-demographic (age range: < 40 years, 40–59 years, 60+ years, gender, employment status: active/inactive), and disease characteristics (time since diagnosis < 10 years / 10–20 years / 20+ years, current treatment: agalsidase alfa, agalsidase beta, migalastat) to ensure items specific to sub-populations were retained.

#### Comprehensibility validity

Participants were asked to confirm whether each item was clear and understandable and if not, were given the possibility of suggesting alternative wording.

#### Completeness validity

Participants were able to provide free comments on potentially missing items or on the PNQ Fabry V1 in general.

#### Internal consistency

An item-to-item correlation was performed to eliminate redundant items and identify independent items to be deleted. Null and high correlation between items were analysed to delete totally independent items and redundancies. When a null correlation between two items occurred, one was deleted based on their performance on other statistical analyses. Those items with a correlation coefficient of > 0.6 were considered highly correlated, thus candidates for grouping with other similar items or if redundant, candidate for deletion.

The Bartlett’s sphericity test was performed to validate the hypothesis that items were not totally independent with each other but not redundant either. A significance test (*p* < 0.05) validated this hypothesis. If the *p*-value was not significant, it meant that the items from the list were too independent and that the selection of items should continue.

A Cronbach alpha test was performed to verify internal consistency. Each time an item was deleted, the Cronbach alpha test was performed, as the resulting score increased, the list approached stability. A value of > 0.7 was set as the limit for stability and homogeneity.

#### Structural validity

Kaiser-Meyer-Olkin (KMO) sampling adequacy measure was performed to confirm a factor analysis could be conducted on the dataset if a score higher than 0.49 was reached.

An exploratory factor analysis (EFA) with Varimax rotation was performed on all items to identify the main factors and to identify item clusters or item outliers based on their load on each factor.

A scree test was performed to summarize a maximum amount of information from the initial item list into key factors whose eigenvalue is greater than 1. We hypothesised that items should have a factor load above 0.4 to be retained. Multiple high factor loads or low factor loads highlighted candidate items to eliminate.

### STEP III: quantitative reliability testing

Participants from step II were invited to participate in step III and additional participants were recruited. Data was collected in the same manner as in Step II.

#### Reliability

The reliability of the questionnaire was validated with a 2-round survey (test and retest) conducted among the same participants. For each participant, there was a minimum of 2 weeks interval between the two rounds. To validate the intrinsic stability, the differences between scores per item and per individual were calculated between the two survey rounds. It was hypothesised that there would be no significant difference between the rounds.

Reliability was demonstrated through several statistical analyses. The difference of mean scores for each item was computed for statistically significance between the test and retest rounds using Student’s T-test. The proportion of participants selecting the option “Does not apply to me” was also computed for statistically significance between the test and retest rounds using Student’s T-test. Additionally, the proportion of “perfect match” was calculated for each item: same score given by one individual between test and retest rounds.

The Pearson’s correlation coefficient was calculated for both test and retest data. A score > 0.7 would indicate a linear relationship between the set of items tested in each survey.

#### Supplementary validation compared with a validated PRO (WHOQOL-BREF)

Due to the disease rarity and the risk of having a sample size too small to detect any significant differences between the test and retest surveys, an additional reliability score was obtained by comparing the PNQ Fabry with a benchmark scale (WHOQOL-BREF). This general quality of life instrument measures physical health, psychological health, social relationships, and environment. This validated, reputable tool has been widely used to measure quality of life in a wide variety of medical conditions. It was chosen for its similarity to the PNQ Fabry to help comparative analysis: both have the same 5-Point Likert scale, a “Does not apply to me” option and same number of items. Furthermore, choosing a general health quality of life scale avoided introducing redundant questions and participant boredom.

All reliability analyses were performed for both the WHOQOL-BREF and PNQ Fabry.

Statistical significance tests were performed to demonstrate if the reliability performance was comparable between the PNQ Fabry and the validated PRO (WHOQOL-BREF). If so, this would provide additional reliability evidence for the PNQ Fabry.

## Results

### STEP I: concept elicitation

#### Literature review

One hundred and ten publications were identified, published between 2004 and 2017 and 31 relevant articles were selected. No Patient-Reported Outcomes instrument addressing the research objectives was found.

#### Concept elicitation interviews and building of the conceptual framework

Eight patients aged between 23 and 68, four males and four females with Fabry disease were interviewed. Three lived in a rural area and five in urban areas. The socioeconomic status was high for three, middle for four and low for one participant. All but three were working, one was a student, one was retired, and one had disability status. Two participants were receiving agalsidase alfa, three agalsidase beta, two migalastat and one was not receiving any treatment but was planning to start in the following 3 months.

The interview verbatim elicited 94 possible items. After eliminating redundant items and nuances, those items that best represented the concepts drawn from the interviews and literature review were retained.

The final conceptual framework consisted of 58 items, grouped into 26 root concepts, which in turn corresponded to five main dimensions; long-term efficacy, impact on daily activities, effectively treated symptoms, impact of the treatment administration, perception of the mode of administration (Additional file 1: Table S1). This 58-item list was named Version 1 PNQ Fabry.

### STEP II: psychometric analysis and cognitive validation

The step II survey data was collected between October 2nd, 2017 and December 12th, 2017. Of the 150 questionnaires distributed to participants, 95 were returned. Two questionnaires were excluded for incompleteness. Therefore, 93 questionnaires without missing values were included in the statistical analysis. Patient associations collected 57 questionnaires (APMF: 47 and VML: 10) and 36 were collected by participating physicians. The population was appropriately heterogeneous in terms of gender (57% were women); age (24.7% aged < 40 vs 46.2% aged 40–59 and 28% aged 60+), family situation (76.3% living with one or more family member), employment status (35.5% working full-time), time since diagnosis (12.9% more than 20 years vs 38.7% 10–20 years vs 44.1% less than 10 years). Forty-three percent of respondents were taking agalsidase beta, 36.6% agalsidase alfa, 11.8% migalastat, 5.4% did not specify their treatment and 3.2% declared not currently taking any treatment. For full details see Additional file 1: Table S2.

Items were reduced according to the combined computed performance on the 5 critical criteria:

#### Content validity

Among the initial list of 58 items, the percentage of participants who rated any given item as “5-very important” ranged from 19.4 to 96.8%. The cumulative percentage of participants who rated any given item “5-very important” or “4-important” ranged from 28 to 100%. Among the final 26-item list, items rated as “5-very important” ranged from 46.2 to 96.8% of participants and the cumulative percentage of patients who rated “5-very important” or “4-important” ranged from 63.4 to 100%.

Among the initial list of 58 items, the percentage of participants who chose the item “Does not apply to me” ranged from 0 to 47.3%. Among the final 26-item, the percentage of participants who chose the item “Does not apply to me” ranged from 0 to 34.4%.

#### Comprehensibility validity

The final 26-item list was well understood: for each item, the percentage of participants who considered that it was clear and understandable ranged from 81.7 to 95.2%.

One hundred and seventy comments were collected in the open fields associated to each item. Their analysis contributed to item deletion, rephrasing and grouping.

#### Completeness validity

The 39 free comments about the PNQ and the list of items in general were considered. No new items were added to the PNQ Fabry, demonstrating that the items covered both general and specific treatment needs and expectations for Fabry patients.

#### Internal consistency

The item-to-item correlation identified 11 null correlations between items and 23 items with a high item-to-item correlation > 0.6 that were considered for redundancy or independence.

The final 26-item list obtained a significance of *p* = 0.041 (< 0.05) on the Bartlett test. This confirmed the consistency of this list of items: each item being neither totally independent nor redundant.

The internal consistency testing showed the 26-item list was stable and relevant with a Cronbach alpha score of 0.845, above the threshold of 0.7.

#### Structural validity

The following analyses demonstrated that the final list of 26 items had a solid structural validity; each item individually contributing to key factors and altogether appropriately covering the topic.

The data set was eligible for an explorative factor analysis (EFA) with a KMO score of 0.722. The scree test determined nine key factors with eigenvalue > 1 explaining 70.8% of the variance. After rotation and selection of most relevant items, each of the 26 items appropriately loaded one of the key factors with a value higher than 0.4.

This final list of 26 items was named PNQ Fabry. This validated version exists in French (Additional file 1: Table S3) however, it has been translated into English for the purposes of this article. (Table 2).

**Table 2:** PNQ Fabry (final version – 26 items)

**How important is it to you that the treatment …**						
*Please tick a box for each statement*	Not at all important	Slightly important	Moderately important	Fairly important	Very important	Does not apply to me
ensures you feel less tired	□	□	□	□	□	□
reduces the pain in your hands and feet	□	□	□	□	□	□
ensures you are less breathless when performing daily activities or with strenuous activities	□	□	□	□	□	□
reduces gastrointestinal disorders (nausea, pain, diarrhoea, constipation)	□	□	□	□	□	□
enables you to tolerate variations with heat and temperature better	□	□	□	□	□	□
reduces the intensity, frequency, or duration of painful attacks	□	□	□	□	□	□
enables you to continue working	□	□	□	□	□	□
enables you to cope with physical exertion better	□	□	□	□	□	□
enables you to live normally, as if you did not have Fabry disease (handicraft, housework, gardening, playing with your children, grandchildren.)	□	□	□	□	□	□
enables you to maintain your social life (work, school, family, friends.)	□	□	□	□	□	□
enables you to travel easily	□	□	□	□	□	□
enables you to have a better quality of life	□	□	□	□	□	□
ensures you are not dependent on other people on a daily basis	□	□	□	□	□	□
enables you to spend time with your family	□	□	□	□	□	□
enables you to stay fit for longer	□	□	□	□	□	□
prevents the onset of heart, kidney, or neurological problems	□	□	□	□	□	□
slows down the deterioration of your organs (kidneys, heart, brain.)	□	□	□	□	□	□
enables you to feel good every day, even on days preceding or following treatment administration	□	□	□	□	□	□
does not cause side effects or adverse effects related to the medication	□	□	□	□	□	□
ensures you do not experience pain and tiredness returning on days before medication is administered	□	□	□	□	□	□
reduces the amount of medication that you are taking	□	□	□	□	□	□
**How important is it to you to have a treatment …**						
*Please tick a box for each statement*	Not at all important	Slightly important	Moderately important	Fairly important	Very important	Does not apply to me
that easily fits into your schedule and lifestyle	□	□	□	□	□	□
that you can take or administer on your own	□	□	□	□	□	□
that is easy to administer	□	□	□	□	□	□
that is administered orally (in tablet or capsule form)	□	□	□	□	□	□
with a short duration of administration	□	□	□	□	□	□

### STEP III: quantitative reliability testing

The survey data was collected between March 6 and May 23, 2018 with an interval of 15 to 30 days between test and retest rounds. Eighty-nine patients participated in the test round. Of these, 76 also participated in the retest round. Thus, only these 76 test and retest questionnaires were included in the statistical analysis. Of those, 42 were collected by the APMF and 10 by the VML association and 24 by the participating physicians. The population was appropriately heterogeneous in terms of socioeconomic, geographic and treatment backgrounds. See Additional file 1: Table S2.

Results demonstrated the PNQ Fabry is highly reliable:

Deviations between scores obtained in both rounds calculated for each item and per individual showed excellent similarity with 72.7% of identical ratings (deviation = 0) between test and retest rounds (Table 3) and 91% only deviated one point above or below (Table 4).

These results were similar to those obtained with the validated benchmark WHOQOL-BREF.

**Table 3:** Reliability assessment: Percentage of identical ratings between test and retest rounds (Deviation =0)

Calculated for each item	PNQ Fabry	WHOQOL-BREF
% Minimal of deviation =0 observed for one item	52.6%	46.1%
% Maximal of deviation =0 observed for one item	93.4%	73.7%
Mean of % deviation =0	72.7%	63.0%

**Table 4:** Reliability assessment: Percentage of ratings with a deviation of + 1 or 0 or − 1 of one same item between test and retest rounds (Deviation = [−1; + 1])

Calculated for each item	PNQ Fabry	WHOQOL-BREF
% Minimal of deviation [−1; + 1] observed for one item	78.9%	73.7%
% Maximal of deviation [−1; + 1] observed for one item	98.7%	97.4%
Mean of % deviation [−1; + 1] observed for all items	90.9%	90.5%

Mean scores obtained by each item at test and retest rounds were consistent. No significant difference was observed at significance level α risk = 0.05 for all items and at significance level α risk = 0.01 for 24 out of 26 items) (Additional file 1: Table S4).

These gaps were comparable to those observed for WHOQOL-BREF (benchmark scale) (Table 5).

**Table 5:** Reliability assessment: Scoring differences between test and retest rounds: mean ratings (5-point scale)

Calculated for each item (rating /5)	PNQ Fabry	WHOQOL-BREF
Minimal difference observed for one item	−0.15	−0.18
Maximal difference observed for one item	0.19	0.16
Sum of absolute values of differences observed in all items	0.34	0.34
Mean of absolute values	0.09	0.06

The analysis of the “Does not apply to me” rating confirmed the PNQ Fabry was relevant with no significant difference α risk =0.01 for all items and comparable to WHOQOL-BREF scale (Table 6 and Additional file 1: Table S5).

**Table 6:** Reliability assessment: Difference of percentage of participants who selected ‘Does not apply to me’ between test and retest rounds

Calculated for each item	PNQ Fabry	WHOQOL-BREF
% Minimal difference observed for one item	−3.95	−2.63
% Maximal difference observed for one item	3.95	5.26
Sum of absolute values of differences observed in all items	7.90	7.89
Mean of absolute values	−0.10	−0.20

Furthermore, the Pearson’s correlation coefficients were close: 0.81 for PNQ Fabry and 0.88 for the WHOLQOL-BREF; and both higher than the threshold of 0.7.

## Discussion

The aim of this research was to develop and validate a self-reported questionnaire to assess treatment expectations for people living with Fabry disease. This is important because now that the molecular mechanisms that cause Fabry disease are better understood, [25] specific treatments have become available. (ERT since 2001 and chaperone molecule since 2016 in the EU / 2003 and 2018 in the US, respectively) [26–28]. This shift in the treatment paradigm has created the opportunity to consider patient specificities and preferences in the treatment decision [1, 29]. As Fabry disease is rare, chronic and varies greatly in presentation and progression, the PNQ Fabry will be a valuable tool for clinicians to understand patient needs, and to refine and adapt treatment modality and intensity accordingly. In line with the current trend towards patient-centric care, this self-reported questionnaire is easy to implement into a long-term care program. Any healthcare professional can suggest that a patient use the questionnaire to identify treatment needs either when treatment changes are required, or at any time deemed necessary [6].

Expert clinicians and patient associations were involved at each step of the PNQ Fabry development to review and endorse research outcomes.

To ensure that PNQ Fabry captured the whole spectrum of patient needs, items were created from open interviews with Fabry patients. In the qualitative and quantitative steps, elicited items were reduced from 94 to 58, then to a final list of 26 items. Particular attention was paid to designing a questionnaire with an appropriate number of items for this population to manage. The psychometric analysis and cognitive validation found that the PNQ Fabry exhibited excellent reliability, internal validity and content validity. Therefore, the 26 items accurately address all themes elicited from patient interviews.

To validate reliability, PNQ Fabry was tested and then retested a minimum of 15 days but no later than 45 days after the test survey. This test and retest interval was determined following FDA guidelines for PRO development, [21], [30] which recommend defining the time interval according to the participant population, disease type, PRO objective and variables to avoid memorisation effect or to capture actual changes.

It was hypothesised that reliability would be shown if there were no significant differences between the two data sets and if the Pearson correlation score was greater than 0.7. The analysis found no significant differences between the two rounds and a Pearson correlation score of 0.81, which demonstrates excellent reliability.

In rare, chronic diseases, where statistically robust samples are difficult to reach, the ISPOR working group [21] recommends also testing the new questionnaire against a validated benchmark PRO to provide an additional set of reference values for comparison purposes. Therefore, numerous HRQoL questionnaires were considered. The WHOQOL-BREF was chosen as it best met the criteria to minimize bias [31]. The WHOQOL-BREF is a validated, frequently used questionnaire and with a different topic to avoid redundancy. Furthermore, this questionnaire is relevant to the patient population, it is validated in French and has a similar length, rating scale and a ‘not concerned’ option. Reliability results of the PNQ Fabry were similar to those obtained with the WHOQOL-BREF.

Patient recruitment was a significant challenge for this research. The rare nature of this disease prevented the cohort from meeting the standard recommended sample size for PRO development. Lacking epidemiological data, the medical community currently estimates the Fabry population in France to be around 600 with 450 treated. Thus, considering this and the chronic, heterogeneous nature of this disease, patient recruitment was key to ensuring that the included population represented the community population and that all needs for all patient profiles, forms and severity levels were considered. This was made possible by building a synergistic collaboration with the two patient associations (APMF and VML) and the expert medical centres to facilitate patient recruitment in order to reach a statistically representative sample. The questionnaire return rates were excellent, demonstrating that the participants were highly-motivated.

Having recruited patients through the patient associations, it’s worth noting that these patients may have scored item importance higher and provided more complete responses to open-ended questions. Members of patient associations may be more dedicated to managing their disease and aware of their needs and expectations. However, this did not impact on the selection of the 26 final items of the PNQ Fabry.

The PNQ Fabry was developed in French. However, validating the PNQ Fabry in other languages and making it a standard instrument for Fabry Disease patient management in many countries would be an important next step.

## Conclusions

Our data suggest the 26-item PNQ Fabry is a reliable and valid self-reported questionnaire to assess patient treatment needs and expectations.

The PNQ Fabry is being used in an ongoing, prospective phase IV clinical study whose primary objective is to cluster patients according to common needs and evaluate treatment benefit at follow up.

## Supplementary information

**Additional file 1: Table S1.** An excerpt from the conceptual tree, illustrating the conceptual framework from which the questionnaire was derived. **Table S2.** Step II and Step III: patient characteristics. **Table S3.** PNQ Fabry (final version – 26 items) (French version)***.*** **Table S4.** Observed differences between the results for test and re-test rounds: reliability assessment – mean ratings*.* **Table S5.** Observation of the differences between the results for test and re-test rounds: reliability assessment – ratings as ‘Does not apply to me’

## Data Availability

The datasets generated and analysed during this study are not publicly available. Researchers may request data access, detailing their reasons for access to Amicus Therapeutics (fdupuissimeon@amicusrx.com) and A + A Research (contact@aplusaresearch.com). Request for access will be reviewed by the Amicus Therapeutics scientific committee. Additional results supporting the conclusions of this article are included in additional file 1.
