# Supplementary material for: Treatment needs and expectations for Fabry disease in France: development of a new Patient Needs Questionnaire
Source: Orphanet J Rare Dis. 2019 Dec 4;14:284. doi: 10.1186/s13023-019-1254-7 (PMC6894302; doi:10.1186/s13023-019-1254-7)
Supplement: Supplementary file 1 — Additional file 1: Table S1. An excerpt from the conceptual tree, illustrating the conceptual framework from which the questionnaire was derived. Table S2. Step II and Step III: patient characteristics. Table S3. PNQ Fabry (final version – 26 items) (French version). Table S4. Observed differences between the results for test and re-test rounds: reliability assessment – mean ratings. Table S5. Observation of the differences between the results for test and re-test rounds: reliability assessment – ratings as ‘Does not apply to me’ [file 13023_2019_1254_MOESM1_ESM.docx]

ADDITIONAL FILE 1

Table S1. An excerpt from the conceptual tree, illustrating the conceptual framework from which the questionnaire was derived.

| Dimension | Root concept | Item |
| --- | --- | --- |
| Long-term efficacy (clinical impacts) | Extended life expectancy | *ensures you live longer* |
|  |  | *stops the progression of Fabry disease* |
|  |  | *slows the progression of Fabry disease* |
|  | Long term organ protection | *slows down the deterioration of your organs (kidneys, heart, brain, etc.)* |
|  |  | *stops the deterioration of your organs (kidneys, heart, brain, etc.)* |
|  |  | *improves the condition of your organs (kidneys, heart, brain, etc.)* |
|  |  | *prevents the onset of cardiac, renal, or neurological problems (stroke, etc.)* |
|  | Project themselves in the future | *enables you to make plans for the future* |
|  |  | *enables you to have good results in your medical examinations* |
|  |  | *enables you to stay fit for longer* |
|  |  | *enables you to have a better quality of life* |
| Direct impact of therapy | Impact on fitness | *enables you to feel good every day* |
|  |  | *ensures you do not have a decrease in fitness on days before medication is administered* |
|  |  | *ensures you do not have a decrease in fitness on days after medication is administered* |
|  |  | *ensures you do not experience pain and tiredness returning on days before medication is administered* |
|  | Impact on daily activities | *ensures you do not reduce your activities or social interactions on days before medication is administered* |
|  |  | *ensures you do not reduce your activities or social interactions on days after medication is administered* |
|  | Side effects | *does not cause side effects or adverse effects related to the medication* |
|  |  | *does not cause excessive sweating* |
|  | Concerns/fear related to the route of administration | *does not cause a feeling of dread before administration of the treatment* |
|  | Impact on concomitant medications | *reduces the amount of medication that you're taking* |
|  | Impact on the number of visits to the hospital | *reduces the number of hospital visits for administering medication* |
| Mode of administration for therapies | Organization due to administration | *that you won’t forget to take* |
|  |  | *that is easy to administer* |
|  |  | *that is fast to administer* |
|  |  | *that requires little or no organization on your part for administration or management* |
|  |  | *that easily fits into your schedule and lifestyle* |
|  | Route of administration | *that is administered orally (in tablet or capsule form)* |
|  |  | *that is administered by infusion* |
|  | Place of administration | *that does not require you to go to hospital and can be administered at home* |
|  | Person administering the treatment | *that is administered by healthcare professionals, such as nurses* |
|  |  | *that you can take or administer on your own* |

Table S2. Step II and Step III: patient characteristics.

|  | **Step II** | | **Step ~~III~~** | |
| --- | --- | --- | --- | --- |
| **Gender** | **Mean age (years)** | **%** | **Mean age (years)** | **%** |
| Male | 45.6 | 41.9 | 50.2 | 36.8 |
| Female | 52.9 | 57.0 | 50.7 | 63.2 |
| Not reported |  | 1.1 |  |  |

|  | **Step II** | **Step III** |
| --- | --- | --- |
| **Age [18-80]** | **%** | **%** |
| < 40 years | 24.7 | 22.4 |
| 40-59 years | 46.2 | 50.0 |
| 60+ years | 28.0 | 27.6 |
| Not reported | 1.1 |  |
| **Region** | **%** | **%** |
| Telephone area code 01 (Paris area) | 19.4 | 18.4 |
| Telephone area code 02 (North-West) | 12.9 | 18.4 |
| Telephone area code 03 (North-East) | 37.6 | 31.6 |
| Telephone area code 04 (South-East) | 16.1 | 17.1 |
| Telephone area code 05 (South-West) | 14.0 | 14.5 |
| **Housing area** | **%** | **%** |
| Urban | 58.1 | 61.3 |
| Peri-urban | 11.8 | 8.0 |
| Rural | 29.0 | 30.7 |
| Not reported | 1.1 |  |
| **Family situation** | **%** | **%** |
| Living alone | 22.6 | 28.9 |
| Living with one or more members of the family/spouse or with roommate | 76.3 | 69.7 |
| in a multi-residence housing (retirement home or other) | 1.1 | 1.3 |
| **Employment status** | **%** | **%** |
| Full-time employment | 35.5 | 38.6 |
| Part-time employment | 10.8 | 6.6 |
| Stay-at-home | 2.2 | 3.9 |
| Unemployed | 8.6 | 6.6 |
| Student | 3.2 | 3.9 |
| Disability | 16.1 | 15.8 |
| Retired | 22.6 | 22.6 |
| Other | 1.1 | 2 |
| **Time since diagnosis** | **%** | **%** |
| < 10 years | 44.1 | 44.7 |
| 10-20 years | 38.7 | 35.5 |
| > 20 years | 12.9 | 19.7 |
| Not reported | 4.3 | 0 |
| **Current Specific Treatment** | **%** | **%** |
| Agalsidase beta (Fabrazyme^®^) | 43.0 | 44.7 |
| Agalsidase alfa (Replagal^®^) | 36.6 | 32.9 |
| Migalastat (Galafold^®^) | 11.8 | 7.9 |
| Specific treatment not specified | 5.4 | 11.8 |
| None | 3.2 | 2.6 |
| **Member of a patient association** | **%** | **%** |
| Yes *(tick all associations you are member of)* | 63.4 | 67.1 |
| - APMF | 53.8 | 55.3 |
| - VML | 9.7 | 13.2 |
| - Other AIRG France, Maladies rares | 2.2 | 2.6 |
| No | 36.6 | 32.9 |

Table S3. PNQ Fabry (final version – 26 items) (French version)

Voici une liste d’affirmations concernant **les attentes des patients vis-à-vis de leur traitement** pour la maladie de Fabry.

Pour chacune de ces attentes, veuillez indiquer **l’importance** que vous leur accordez en cochant la case correspondante.

Si une affirmation ne vous concerne pas, par exemple parce que vous ne travaillez pas, veuillez cocher « Ne me concerne pas »

Si vous ne savez pas comment répondre, choisissez la réponse la plus proche de votre situation.

**Dans quelle mesure est-il important pour vous que le traitement permette de/d’…**

| *Merci de cocher une case pour chaque affirmation* | **Pas du tout important** | **Un peu important** | **Moyennement important** | **Assez important** | **Très important** | *Ne me concerne pas* |
| --- | --- | --- | --- | --- | --- | --- |
| être moins fatigué(e) | ☐ | ☐ | ☐ | ☐ | ☐ | ☐ |
| réduire les douleurs dans les mains et les pieds | ☐ | ☐ | ☐ | ☐ | ☐ | ☐ |
| être moins essoufflé(e) au quotidien et à l'effort | ☐ | ☐ | ☐ | ☐ | ☐ | ☐ |
| réduire les problèmes gastro-intestinaux (nausées, douleurs, diarrhée, constipation) | ☐ | ☐ | ☐ | ☐ | ☐ | ☐ |
| mieux supporter la chaleur et les variations de température | ☐ | ☐ | ☐ | ☐ | ☐ | ☐ |
| réduire l'intensité, la fréquence ou la durée des crises douloureuses | ☐ | ☐ | ☐ | ☐ | ☐ | ☐ |
| continuer à travailler | ☐ | ☐ | ☐ | ☐ | ☐ | ☐ |
| être plus résistant(e) à l'effort | ☐ | ☐ | ☐ | ☐ | ☐ | ☐ |
| vivre normalement, comme si vous n'aviez pas la maladie de Fabry (bricolage, jardinage, jouer avec vos enfants/petits-enfants, entretien de la maison etc.) | ☐ | ☐ | ☐ | ☐ | ☐ | ☐ |
| maintenir votre vie sociale (travail, scolarité, famille, amis, etc.) | ☐ | ☐ | ☐ | ☐ | ☐ | ☐ |
| voyager facilement | ☐ | ☐ | ☐ | ☐ | ☐ | ☐ |
| avoir une meilleure qualité de vie | ☐ | ☐ | ☐ | ☐ | ☐ | ☐ |
| ne pas être dépendant(e) des autres au quotidien | ☐ | ☐ | ☐ | ☐ | ☐ | ☐ |
| pouvoir passer du temps avec votre famille | ☐ | ☐ | ☐ | ☐ | ☐ | ☐ |
| vous maintenir en forme plus longtemps | ☐ | ☐ | ☐ | ☐ | ☐ | ☐ |
| éviter l'apparition de problèmes cardiaques, rénaux ou neurologiques | ☐ | ☐ | ☐ | ☐ | ☐ | ☐ |
| ralentir la dégradation de vos organes (reins, cœur, cerveau, etc.) | ☐ | ☐ | ☐ | ☐ | ☐ | ☐ |
| être en forme tous les jours même les jours qui précèdent et qui suivent l'administration du traitement | ☐ | ☐ | ☐ | ☐ | ☐ | ☐ |
| ne pas souffrir d'effets secondaires ou indésirables liés au traitement | ☐ | ☐ | ☐ | ☐ | ☐ | ☐ |
| ne pas voir revenir les douleurs et la fatigue les jours qui précèdent l'administration du traitement | ☐ | ☐ | ☐ | ☐ | ☐ | ☐ |
| réduire le nombre de médicaments que vous prenez | ☐ | ☐ | ☐ | ☐ | ☐ | ☐ |

**Dans quelle mesure est-il important pour vous d’avoir un traitement…**

| *Merci de cocher une case pour chaque affirmation* | **Pas du tout important** | **Un peu important** | **Moyennement important** | **Assez important** | **Très important** | *Ne me concerne pas* |
| --- | --- | --- | --- | --- | --- | --- |
| qui s'intègre facilement dans votre emploi du temps et votre rythme de vie | ☐ | ☐ | ☐ | ☐ | ☐ | ☐ |
| que vous pouvez prendre ou vous administrer seul | ☐ | ☐ | ☐ | ☐ | ☐ | ☐ |
| dont l'administration est facile | ☐ | ☐ | ☐ | ☐ | ☐ | ☐ |
| administré par voie orale (sous forme de comprimé, gélule) | ☐ | ☐ | ☐ | ☐ | ☐ | ☐ |
| dont la durée d'administration est courte | ☐ | ☐ | ☐ | ☐ | ☐ | ☐ |

Table S4. Observed differences between the results for test and re-test rounds: reliability assessment – mean ratings

| PNQ FABRY | 1 | 2 | 3 | 4 | 5 | 6 | 7 | 8 | 9 | 10 | 11 | 12 | 13 | 14 | 15 | 16 | 17 | 18 | 19 | 20 | 21 | 22 | 23 | 24 | 25 | 26 |
| --- | --- | --- | --- | --- | --- | --- | --- | --- | --- | --- | --- | --- | --- | --- | --- | --- | --- | --- | --- | --- | --- | --- | --- | --- | --- | --- |
| **Mean score Test** | 4.31 | 4.54 | 4.68 | 4.68 | 4.72 | 4.80 | 4.70 | 4.96 | 4.86 | 4.68 | 4.65 | 4.28 | 4.62 | 4.64 | 4.91 | 4.30 | 4.81 | 4.64 | 4.55 | 4.60 | 4.82 | 4.59 | 4.41 | 4.56 | 4.39 | 4.38 |
| **Mean score Re-test** | 4.49 | 4.60 | 4.82 | 4.79 | 4.82 | 4.86 | 4.78 | 4.95 | 4.84 | 4.71 | 4.68 | 4.43 | 4.70 | 4.81 | 4.93 | 4.45 | 4.82 | 4.83 | 4.67 | 4.65 | 4.76 | 4.68 | 4.33 | 4.41 | 4.35 | 4.49 |
| **Test re-test Gap** | 0.18 | 0.06 | 0.14 | 0.11 | 0.10 | 0.05 | 0.08 | -0.01 | -0.02 | 0.03 | 0.03 | 0.16 | 0.08 | 0.17 | 0.03 | 0.15 | 0.01 | 0.19 | 0.12 | 0.05 | -0.06 | 0.10 | -0.08 | -0.15 | -0.04 | 0.11 |
| **T-Test of significance** | 1.25 | 0.51 | 1.23 | 0.99 | 1.26 | 0.73 | 0.92 | 0.32 | 0.26 | 0.29 | 0.31 | 1.04 | 0.56 | 1.84* | 0.57 | 1.05 | 0.19 | 1.82* | 1.05 | 0.38 | 0.50 | 0.97 | 0.47 | 1.02 | 0.22 | 0.88 |

*All results not significant (significance level alpha risk at 1%), except for #14 and #18:*

** Not significant (significance level alpha risk at 5%)*

| **WHOQOL-BREF** | 1 | 2 | 3 | 4 | 5 | 6 | 7 | 8 | 9 | 10 | 11 | 12 | 13 | 14 | 15 | 16 | 17 | 18 | 19 | 20 | 21 | 22 | 23 | 24 | 25 | 26 |
| --- | --- | --- | --- | --- | --- | --- | --- | --- | --- | --- | --- | --- | --- | --- | --- | --- | --- | --- | --- | --- | --- | --- | --- | --- | --- | --- |
| **Mean score Test** | 3.43 | 2.95 | 2.93 | 3.83 | 3.57 | 3.54 | 3.62 | 3.47 | 3.73 | 3.09 | 3.64 | 3.51 | 3.71 | 3.43 | 3.96 | 2.83 | 3.24 | 3.33 | 3.25 | 3.72 | 2.95 | 3.72 | 4.08 | 4.09 | 4.07 | 2.78 |
| **Mean score Re-test** | 3.41 | 2.92 | 3.06 | 3.89 | 3.48 | 3.36 | 3.57 | 3.59 | 3.77 | 3.20 | 3.67 | 3.53 | 3.67 | 3.34 | 3.95 | 2.78 | 3.25 | 3.49 | 3.32 | 3.66 | 2.97 | 3.63 | 4.03 | 4.13 | 4.00 | 2.81 |
| **Test re-test Gap** | -0.03 | -0.03 | 0.13 | 0.06 | -0.09 | -0.18 | -0.05 | 0.13 | 0.04 | 0.11 | 0.03 | 0.02 | -0.04 | -0.09 | -0.01 | -0.05 | 0.01 | 0.16 | 0.07 | -0.06 | 0.02 | -0.09 | -0.05 | 0.04 | -0.07 | 0.03 |
| **T-Test of significance** | 0.18 | 0.16 | 0.67 | 0.27 | 0.53 | 0.78 | 0.30 | 0.69 | 0.19 | 0.58 | 0.14 | 0.10 | 0.23 | 0.45 | 0.08 | 0.31 | 0.04 | 0.75 | 0.46 | 0.43 | 0.08 | 0.57 | 0.34 | 0.22 | 0.39 | 0.16 |

*All results not significant (significance level alpha risk at 1%).*

Regarding the PNQ Fabry, the differences for mean scores between both test rounds were not significant for all items (significance level alpha risk at 1% for 24 out of 26 items, and at 5% for 2 items).

Table S5. Observation of the differences between the results for test and re-test rounds: reliability assessment – ratings as ‘Does not apply to me’

| PNQ FABRY | 1 | 2 | 3 | 4 | 5 | 6 | 7 | 8 | 9 | 10 | 11 | 12 | 13 | 14 | 15 | 16 | 17 | 18 | 19 | 20 | 21 | 22 | 23 | 24 | 25 | 26 |
| --- | --- | --- | --- | --- | --- | --- | --- | --- | --- | --- | --- | --- | --- | --- | --- | --- | --- | --- | --- | --- | --- | --- | --- | --- | --- | --- |
| **% NA**  **Test** | 6.6% | 2.6% | 30.3% | 9.2% | 1.3% | 0.0% | 2.6% | 1.3% | 0.0% | 13.2% | 1.3% | 9.2% | 13.2% | 11.8% | 2.6% | 3.9% | 2.6% | 5.3% | 2.6% | 3.9% | 13.2% | 1.3% | 6.6% | 1.3% | 11.8% | 2.6% |
| **% NA**  **Re-test** | 9.2% | 1.3% | 32.9% | 6.6% | 3.9% | 0.0% | 2.6% | 0.0% | 2.6% | 9.2% | 0.0% | 5.3% | 13.2% | 7.9% | 1.3% | 3.9% | 2.6% | 7.9% | 3.9% | 2.6% | 17.1% | 3.9% | 3.9% | 1.3% | 10.5% | 3.9% |
| **Test Re-test Gap** | 2.63 | -1.32 | 2.63 | -2.63 | 2.63 | 0.00 | 0.00 | -1.32 | 2.63 | -3.95 | -1.32 | -3.95 | 0.00 | -3.95 | -1.32 | 0.00 | 0.00 | 2.63 | 1.32 | -1.32 | 3.95 | 2.63 | -2.63 | 0.00 | -1.32 | 1.32 |
| **T-Test of significance** | 0.60 | 0.58 | 0.35 | 0.60 | 1.01 | 0.00 | 0.00 | 1.00 | 1.42 | 0.77 | 1.00 | 0.94 | 0.00 | 0.82 | 0.58 | 0.00 | 0.00 | 0.65 | 0.45 | 0.45 | 0.68 | 1.01 | 0.73 | 0.00 | 0.26 | 0.45 |

*All results not significant (significance level alpha risk at 1%).*

*NA, not applicable (= Does not apply to me)*

| **WHOQOL-BREF** | **1** | **2** | **3** | **4** | **5** | **6** | **7** | **8** | **9** | **10** | **11** | **12** | **13** | **14** | **15** | **16** | **17** | **18** | **19** | **20** | **21** | **22** | **23** | **24** | **25** | **26** |
| --- | --- | --- | --- | --- | --- | --- | --- | --- | --- | --- | --- | --- | --- | --- | --- | --- | --- | --- | --- | --- | --- | --- | --- | --- | --- | --- |
| **% NA**  **Test** | 0.0% | 0.0% | 5.3% | 7.9% | 1.3% | 17.1% | 0.0% | 1.3% | 1.3% | 0.0% | 1.3% | 1.3% | 0.0% | 0.0% | 1.3% | 0.0% | 2.6% | 27.6% | 1.3% | 1.3% | 14.5% | 0.0% | 0.0% | 0.0% | 3.9% | 0.0% |
| **% NA**  **Re-test** | 0.0% | 0.0% | 10.5% | 5.3% | 1.3% | 15.8% | 0.0% | 0.0% | 2.6% | 0.0% | 0.0% | 0.0% | 0.0% | 0.0% | 0.0% | 0.0% | 0.0% | 30.3% | 1.3% | 0.0% | 11.8% | 0.0% | 0.0% | 0.0% | 1.3% | 3.9% |
| **Test Re-test Gap** | 0.00 | 0.00 | 5.26 | -2.63 | 0.00 | -1.32 | 0.00 | -1.32 | 1.32 | 0.00 | -1.32 | -1.32 | 0.00 | 0.00 | -1.32 | 0.00 | -2.63 | 2.63 | 0.00 | -1.32 | -2.63 | 0.00 | 0.00 | 0.00 | -2.63 | 3.95 |
| **T-Test of significance** | 0.00 | 0.00 | 1.20 | 0.65 | 0.00 | 0.22 | 0.00 | 1.00 | 0.58 | 0.00 | 1.00 | 1.00 | 0.00 | 0.00 | 1.00 | 0.00 | 1.42 | 0.36 | 0.00 | 1.00 | 0.48 | 0.00 | 0.00 | 0.00 | 1.01 | 1.75 |

*All results not significant (significance level alpha risk at 1%).*

*NA, not applicable (= Does not apply to me)*

Regarding the PNQ Fabry, differences for ratings as ‘Does not apply to me’ were not significant between both test rounds (significance level alpha risk at 1%), for all items.
